# Quantitative Analysis of Inflammation in Orbital Fat of Thyroid-associated Ophthalmopathy Using MRI Signal Intensity

**DOI:** 10.1038/s41598-017-17257-6

**Published:** 2017-12-04

**Authors:** Tomoaki Higashiyama, Maki Iwasa, Masahito Ohji

**Affiliations:** Department of Ophthalmology, Shiga University of Medical Science, Otsu, Japan

## Abstract

We quantitatively analyzed inflammation in orbital fat from cases of thyroid-associated ophthalmopathy (TAO) using short-tau inversion recovery (STIR) sequences from magnetic resonance imaging. The signal intensity ratios (SIRs) of orbital fat as well as the superior rectus (SR), inferior rectus (IR), lateral rectus (LR), medial rectus (MR), and superior oblique (SO) muscles on STIR images were measured in 70 eyes from 70 treatment-naive TAO patients (active TAO group, 19 patients; inactive TAO group, 51 patients) and 20 eyes from 20 controls. The mean SIR in the active TAO group was significantly higher than that in the inactive TAO group and controls (*P* < 0.001). The SIR of orbital fat in all TAO patients was significantly (*P* < 0.001) positively correlated with that of the extraocular muscles: SR (*r* = 0.64), IR (*r* = 0.55), LR (*r* = 0.58), MR (*r* = 0.71), and SO (*r* = 0.65). The SIR of orbital fat had a significant positive correlation with the CAS (*r* = 0.53, *P* < 0.001). The measurements of SIRs in orbital fat may be useful in evaluating the activity in tissues of TAO patients.

## Introduction

Thyroid-associated ophthalmopathy (TAO) is the most prevalent extra-thyroidal manifestation of thyroid disease^1^. TAO is an immune-mediated inflammatory disease in which the levels of anti-thyroid antibodies are elevated. The disease causes increased orbital fat and enlarged extraocular muscles in the orbit^1,2^. Some clinical signs and symptoms of TAO arise from enlarged orbital tissues^2–7^. TAO has previously been classified as either the predominantly increased orbital fat type or predominantly enlarged extraocular muscle type^5,8^. Younger patients tend to have increased orbital fat, whereas older patients are more likely to have enlarged extraocular muscles^2^.

We previously reported the quantitative evaluation of inflammation in the extraocular muscles of active TAO patients using short-tau inversion recovery (STIR) sequences of magnetic resonance imaging (MRI)^9^. Inflammation can be quantitatively evaluated in STIR images because the high signal intensity indicates edema caused by inflammation in extraocular muscles^10–16^. Our previous report demonstrated that the pretreatment signal intensity in the extraocular muscles of TAO patients was significantly higher than that in controls by MRI^9^. The signal intensity after methylprednisolone pulse therapy was significantly lower than the pretreatment intensity. In addition, the signal intensity in patients with TAO was significantly correlated with the clinical activity score (CAS). Another study also obtained similar findings^15^.

However, inflammation in the orbital fat of TAO patients has not been studied quantitatively, which has left some question unanswered, such as the comparison of the signal intensities in orbital fat among active TAO patients, inactive TAO patients, and controls as well as the correlation of the inflammation in orbital fat with the inflammation in the extraocular muscles, CAS, and patient’s age.

Thus, we investigated the signal intensity of orbital fat in TAO patients and controls using the MRI STIR sequences.

## Results

### Comparison of the orbital fat signal intensity ratios (SIRs) among the active TAO group, inactive TAO group, and controls

The active (CAS ≥ 3/7) TAO group included 19 patients, and the inactive TAO group included 51 patients. The mean CASs were 3.74 ± 0.73 (range, 3–5) in the inactive TAO group and 1.00 ± 0.85 (range, 0–2) in the inactive TAO group.

**Figure 1:**
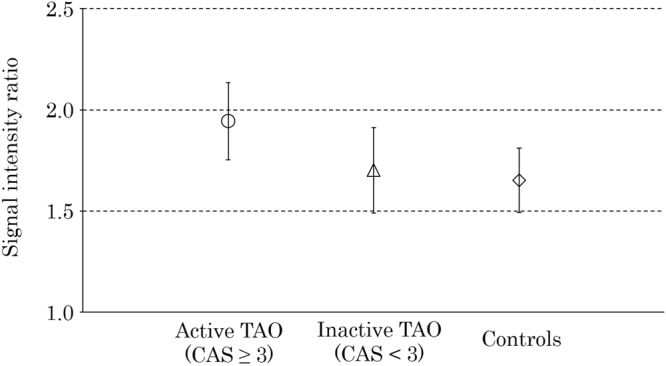
Comparison of the orbital fat signal intensity ratio (SIR) among the active thyroid-associated ophthalmopathy (TAO) group, inactive TAO group, and controls. The plots and bars of the active TAO group (circle), inactive TAO group (triangle) and controls (diamond) indicate the mean and standard deviation. The mean SIR in the active TAO group was significantly higher than that in the inactive TAO group and controls (*P* < 0.001). The mean SIR in the inactive TAO group was not significantly higher than that in the controls (*P* = 0.62).

The mean orbital fat SIRs were 1.94 ± 0.19 in the active TAO group, 1.70 ± 0.21 in the inactive TAO group, and 1.65 ± 0.16 in the controls. The mean SIR in the active TAO group was significantly higher than that in the inactive TAO group and controls (*P* < 0.001) (Fig. 1, Table 1). The mean SIR in the inactive TAO patients was not significantly higher than that in the controls (*P* = 0.62). Table 1 also shows the comparison of the extraocular muscle SIRs among the groups.

**Table 1:** Comparison among the signal intensity ratios of orbital fat in the active thyroid-associated ophthalmopathy (TAO) group, inactive TAO group, and controls.

	Active TAO	Inactive TAO	Controls	P value		
				Active TAO vs. Inactive TAO	Active TAO vs. Controls	Inactive TAO vs. Controls
FAT	1.94 ± 0.19	1.70 ± 0.21	1.65 ± 0.16	<0.001	<0.001	0.62
SR	2.56 ± 0.70	1.78 ± 0.62	1.08 ± 0.25	<0.001	<0.001	<0.001
IR	2.76 ± 0.72	2.03 ± 0.58	1.32 ± 0.28	<0.001	<0.001	<0.001
LR	2.14 ± 0.45	1.75 ± 0.32	1.34 ± 0.18	<0.001	<0.001	<0.001
MR	2.61 ± 0.53	1.91 ± 0.36	1.47 ± 0.24	<0.001	<0.001	<0.001
SO	2.14 ± 0.47	1.58 ± 0.30	1.26 ± 0.22	<0.001	<0.001	<0.001

TAO = thyroid-associated ophthalmopathy; FAT = orbital fat, SR = superior rectus; IR = inferior rectus; LR = lateral rectus; MR = medial rectus; SO = superior oblique.

### Correlations between the orbital fat SIRs and the extraocular muscle SIRs

**Figure 2:**
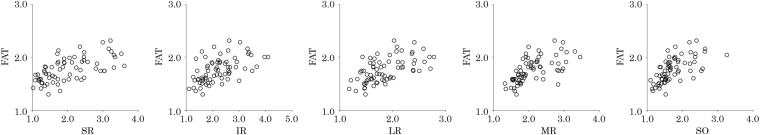
Correlation between the signal intensity ratio (SIR) of the orbital fat and those of extraocular muscles. The orbital fat SIR showed significant (*P* < 0.001) positive correlations with those of extraocular muscles: SR (*r* = 0.64), IR (*r* = 0.55), LR (*r* = 0.58), MR (*r* = 0.71), and SO (*r* = 0.65). FAT = orbital fat; SR = superior rectus; IR = inferior rectus; LR = lateral rectus; MR = medial rectus; SO = superior oblique.

The mean SIRs in all TAO patients were 1.99 ± 0.73, 2.23 ± 0.69, 1.86 ± 0.39, 2.10 ± 0.51 and 1.73 ± 0.43 in the superior rectus (SR), inferior rectus (IR), lateral rectus (LR), medial rectus (MR), and superior oblique (SO) muscles, respectively. The orbital fat SIRs showed significant (*P* < 0.001) positive correlations with those of the extraocular muscles: SR (*r* = 0.64), IR (*r* = 0.55), LR (*r* = 0.58), MR (*r* = 0.71) and SO (*r* = 0.65) (Fig. 2, Table 2).

**Table 2:** Correlations between orbital fat and extraocular muscle signal intensity ratios (SIRs).

	r	*P* value
SR	0.64	<0.001
IR	0.55	<0.001
LR	0.58	<0.001
MR	0.71	<0.001
SO	0.65	<0.001

FAT = orbital fat, SR = superior rectus; IR = inferior rectus; LR = lateral rectus; MR = medial rectus; SO = superior oblique.

### Correlation between orbital fat SIRs and the CAS

**Figure 3:**
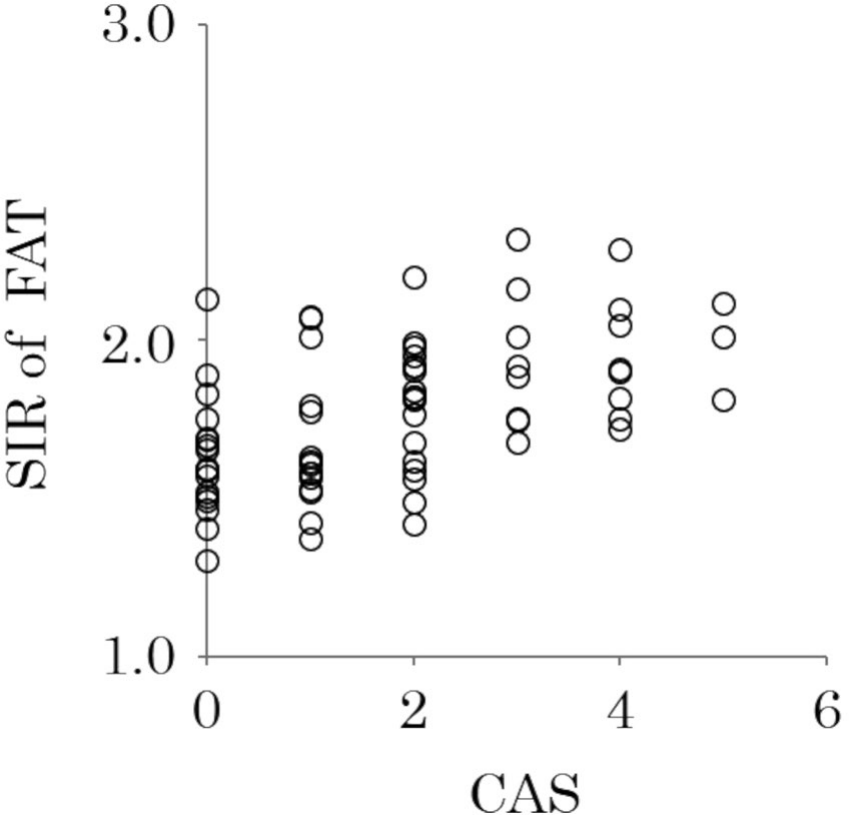
Correlation between the orbital fat signal intensity ratio (SIR) and clinical activity scores. The orbital fat SIR showed a significant positive correlation with the Clinical Activity Score (CAS) (*r* = 0.53, *P* < 0.001). SIR of FAT = signal intensity ratio of orbital fat.

The correlation between the orbital fat SIR and the CAS was analyzed. The mean value in all TAO patients was 1.74 ± 1.47. The orbital fat SIR showed a significant positive correlation with the CAS (*r* = 0.53, *P* < 0.001) (Fig. 3).

### Correlations between orbital fat SIRs and age

**Figure 4:**
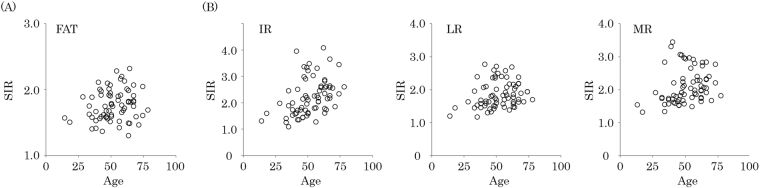
Correlation of age with the signal intensity ratio (SIR) of orbital fat and extraocular muscles. The orbital fat SIR did not show a significant correlation with age (*r* = 0.19, *P* = 0.11) (**A**). The SIRs of some extraocular muscles showed significant positive correlations with age: IR (*r* = 0.42, *P* < 0.001), LR (*r* = 0.24, *P* = 0.047) and MR (*r* = 0.36, *P* = 0.002) (**B**). FAT = orbital fat; IR = inferior rectus; LR = lateral rectus; MR = medial rectus.

A significant correlation was not observed between the orbital fat SIR and age (*r* = 0.19, *P* = 0.11) (Fig. 4A). Very weak significant positive correlations were observed between the SIRs of some of the extraocular muscles and age: IR (*r* = 0.41, *P* < 0.001), LR (*r* = 0.24, *P* = 0.047), MR (*r* = 0.36, *P* = 0.002) (Fig. 4B).

The mean ages were 41.5 ± 8.6 years (range 14–50 years) in the young group (36 patients) and 62.6 ± 6.2 years (range 51–78 years) in the old group (34 patients). Very weak significant positive correlations were observed between the SIRs of some of the extraocular muscles and age in the young group: SR (*r* = 0.46, *P* = 0.005), IR (*r* = 0.38, *P* = 0.23), LR (*r* = 0.34, *P* = 0.04) and MR (*r* = 0.34, *P* = 0.04). However, a significant correlation was not observed between the extraocular muscles SIRs and age in the old group (Table 3). Significant correlations were not observed between the orbital fat SIR and age in either group.

**Table 3:** Correlations between orbital fat and extraocular muscle signal intensity ratios (SIRs) and age.

	Young group		Old group	
	r	*P* value	r	*P* value
FAT	0.28	0.10	−0.15	0.39
SR	0.46	0.05	−0.14	0.42
IR	0.38	0.02	0.09	0.60
LR	0.34	0.04	−0.17	0.33
MR	0.34	0.04	−0.16	0.37
SO	0.32	0.06	0.11	0.55

FAT = orbital fat, SR = superior rectus; IR = inferior rectus; LR = lateral rectus; MR = medial rectus; SO = superior oblique.

## Discussion

The mean SIR in the active TAO group was significantly higher than that in the inactive TAO group and controls in this study. Chen *et al*. reported a comparison of macrophage infiltration into orbital fat between TAO patients and controls^17^. These authors showed that macrophage infiltration into orbital fat was greater in TAO patients than in controls. Histological examination to quantitatively evaluate inflammation in the orbital fat in a clinical setting, however, is almost impossible for many TAO patients. Hence, the noninvasive use of MRI STIR sequences to quantitatively evaluate the signal intensity of orbital fat inflammation may be useful in TAO patients. The current study revealed that orbital fat inflammation may develop due to increased disease activity in TAO patients.

A significant positive correlation was observed between the SIR of orbital fat and that of the extraocular muscles in this study. Other studies previously reported that TAO patients could be classified as having predominantly increased orbital fat or predominantly enlarged extraocular muscles^5,8^. Hiromatsu *et al*. investigated the cytokine profiles of orbital fat and extraocular muscle tissues in TAO patients^8^. These authors reported that the cytokine profiles were different between these two tissue sources. Kuriyan *et al*. investigated the effect of cyclooxygenase inhibitors on adipogenesis and proliferation of orbital fibroblasts in the two TAO subtypes (the predominantly fat compartment enlargement type and predominantly extraocular muscle enlargement type)^5^. Orbital fibroblasts were isolated and established from TAO patients undergoing orbital decompression. The authors reported that the effect of cyclooxygenase inhibitors was different for the two subtypes because the cyclooxygenase inhibitors significantly decreased proliferation and adipogenesis in the orbital fibroblasts of patients with the predominantly extraocular muscle enlargement type but not the predominantly fat compartment enlargement type. In contrast, our study showed that the orbital fat and extraocular muscle SIRs were significantly correlated. We showed that the orbital fat inflammation might develop in conjunction with that in extraocular muscles, although the pathological pathways in these tissues might be different.

The orbital fat SIR showed a significant positive correlation with the CAS in this study. We previously reported a significant positive correlation between the extraocular muscle SIRs and CAS in TAO patients^9^. Another study also reported that a significant positive correlation was found between the extracocular muscle SIRs and the Mourits score, which is the basis for the CAS in TAO patients^14^. Our current study revealed that the orbital fat SIR was also correlated with the CAS.

A significant correlation was not found between the orbital fat SIR and age, whereas a very weak significant positive correlation was observed between some of the extraocular muscle SIRs and age. In other review articles, younger patients were more likely to exhibit increased orbital fat without extraocular muscle enlargement, whereas older patients were more prone to exhibit severe extraocular muscle enlargement^2,18,19^. Murakami *et al*. investigated the relationship between age and extraocular muscle enlargement by CT in TAO patients^20^. The frequency of extraocular muscle enlargement was correlated with age in older patients, which agreed with our results. To the best of our knowledge, the relationship between age and orbital fat inflammation has not been previously reported. The current study showed that the SIR of orbital fat did was not significantly correlated with age. Inflammation in the fat might not be strongly affected by age, unlike that in the extraocular muscles.

The present study has several limitations. First, the sample size was small, with only 70 patients enrolled. Second, the mean CAS was low. It is known that the clinical signs of TAO differ among races, and the CAS tends to be low in Japanese patients with TAO^21^, which could be the reason for the low CAS observed in this study.

In conclusion, measuring the orbital fat SIR may be useful for evaluating disease activity in TAO patients.

## Methods

### Subjects

The institutional review board of Shiga University of Medical Science approved this retrospective study. The study adhered to the tenets of the Declaration of Helsinki. Each patient provided written informed consent.

In total, 70 eyes from 70 patients with TAO (52 female, 18 male; mean ± SD age 51.8 ± 13.0 years, range 14–78 years) and 20 eyes from 20 volunteer controls (4 female, 16 male; mean age 36.8 ± 11.3 years, range 20–54 years) were included in this study at the Department of Ophthalmology, Shiga University of Medical Science Hospital, from April 2008 to December 2016. The eye in which the mean orbital fat SIR was higher was selected as representative of the patient, and the right eye was selected as representative of the control. Only treatment-naive TAO patients who had a high level of serum thyroid autoantibodies were included. The normal serum thyroid autoantibody ranges are as follows: thyrotropin receptor antibody determined by the first and second generation assays (first normal: 0–15%, second normal: 0–0.9 IU/L), thyroid stimulating antibody (normal 0–120%), thyroid peroxidase antibody (normal 0–15.9 IU/mL), and thyroglobulin antibody (normal 0–27.9 IU/mL). If patients had an orbital disease or an inflammatory disease in the orbit, they were excluded.

### Signal intensity measurements

All patients were subjected to orbital MRI with a standard head coil. The MRI was performed on a 3.0-tesla MRI unit (Achieva 3.0 T Quasar Dual; Royal Philips Electronics, Amsterdam, The Netherlands).

**Figure 5:**
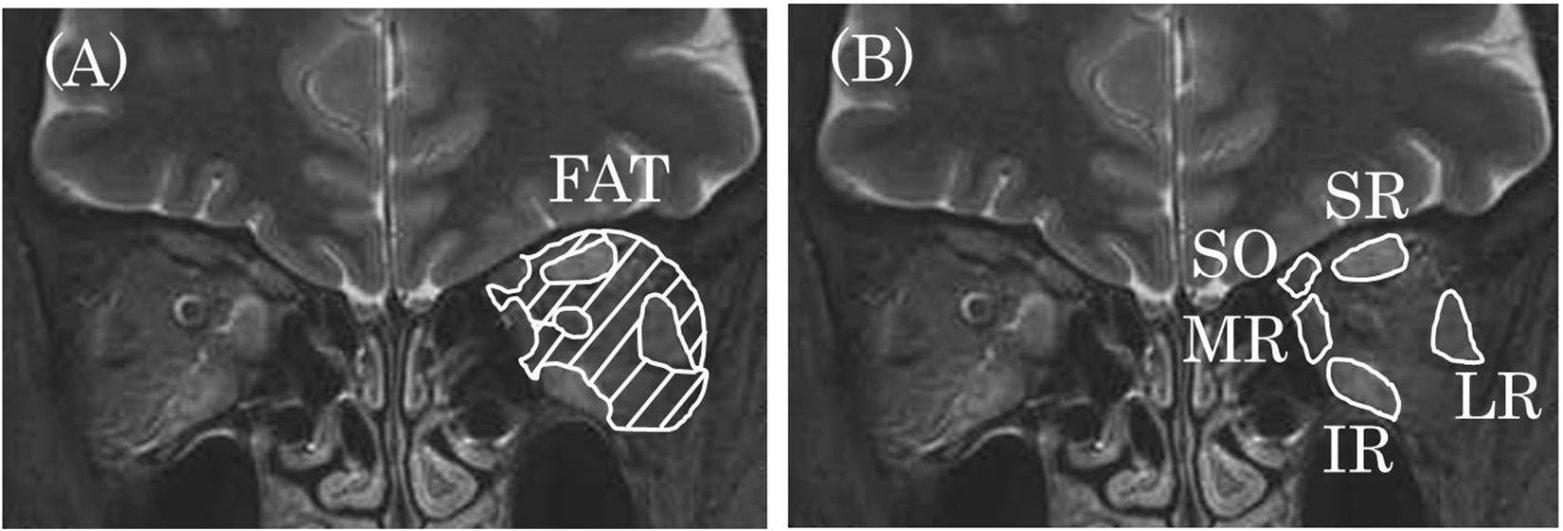
Measurement of signal intensities of orbital fat and extraocular muscles. The signal intensities of orbital fat (**A**) and each muscle (**B**) were measured, as shown, in the left orbit and brain. FAT = orbital fat, SR = superior rectus; IR = inferior rectus; LR = lateral rectus; MR = medial rectus; SO = superior oblique.

The signal intensities of the orbital fat and the SR, IR, LR, MR, and SO muscles were measured in the STIR and T2-weighted images (Fig. 5). The regions of interest (ROIs) were drawn around the tissues on coronal T2-weighted images. The drawn ROIs were superimposed on the STIR images at the same level, and the signal intensities were measured.

To standardize the signal intensity in each tissue, the signal intensity of brain white matter (WM) on STIR images was measured as the “standard” tissue, which was similar to the protocol described in our previous studies^9,16^. The SIRs between the orbital fat or extraocular muscles and the WM of the same slice was calculated. The highest value among the mean SIRs of three consecutive slices in each tissue was considered representative of the SIR for that tissue.

### Assessment of CAS

The CAS (maximum score 7 points) was measured in all TAO patients. The CAS consists of seven findings: spontaneous retrobulbar pain; pain on attempted up or down gaze; redness of the conjunctiva; redness of the eyelids; inflammation of the caruncle and/or plica; swelling of the eyelids; and conjunctival edema^22,23^. Patients with CAS ≥ 3/7 were considered as having active TAO^22^. TAO patients were divided into two groups (the active CAS group and inactive CAS group) in the comparison analysis.

### Statistical analysis

All statistical analyses were performed using SPSS Statistics 22 software (IBM, Armonk, NY, USA). The normality of the numerical variables was evaluated using the Shapiro–Wilk test. The Tukey HSD test was used to compare the SIRs among the active TAO group, inactive TAO group, and controls. Pearson’s product–moment correlation coefficient or Spearman’s rank correlation coefficient were used to analyze the correlations between the orbital fat SIRs and those of extraocular muscles and the correlations between the SIRs and CAS and age in all TAO patients. The values are expressed as the mean ± SD. A *P* value of less than 0.05 indicated statistical significance.
